# Tracing Poly(Vinyl Acetate) Emulsions by Infrared and Raman Spectroscopies: Identification of Spectral Markers

**DOI:** 10.3390/polym13213609

**Published:** 2021-10-20

**Authors:** Susana França De Sá, Carolina Viana, Joana Lia Ferreira

**Affiliations:** LAQV-REQUIMTE, Department of Conservation and Restoration, NOVA School of Science and Technology, Universidade Nova de Lisboa, 2829-516 Caparica, Portugal; c.viana@campus.fct.unl.pt

**Keywords:** poly(vinyl acetate), aqueous vinyl emulsions, plastic paints formulations, infrared spectroscopy, Raman spectroscopy, spectral markers

## Abstract

Vinyl emulsions started to be used by artists in paintings at least since the early 1960s, being now present in several artworks worldwide. However, different vinyl formulations can result in distinct behaviours over time, and if some artworks are currently showing a good condition, others already show damages due to the use of compositions more susceptible to degradation. For this reason, it is fundamental to identify the main components in the vinyl acetate-based (VAc-based) emulsion. This work focuses on the molecular study of VAc-based emulsions by infrared and Raman spectroscopies. It aims at deepening the knowledge on the variability of the composite formulation and on the identification of characteristic bands and spectral profiles (identified as spectral markers) for both polymer and additives. To this end, a broad set of vinyl emulsions was gathered, including reference materials, historical commercial brands in use by Portuguese artists, and commercial brands collected from industrial companies. The entire set includes vinyl homopolymers produced for the purpose of the study and known formulations of vinyl homopolymers and copolymers, with and without plasticisers, according to technical data sheets and previous studies. Furthermore, unknown formulations have been included to validate the usefulness of the identified spectral markers. This set has been studied in the form of solid films deposited in glass slides by infrared spectroscopy in attenuated total reflection mode (ATR-FTIR) and micro-Raman spectroscopy (µ-Raman), both conducted in situ. As conclusions, the combined use of ATR-FTIR and µ-Raman proved to be very useful as different spectral markers were detected by each technique, confirming their complementarity. Besides the clear identification of vinyl acetate-based emulsions by both techniques, it was also possible to suggest spectral markers for the copolymerisation of vinyl acetate with vinyl versatate by µ-Raman, the stabilisation of the emulsion with poly(vinyl alcohol) by ATR-FTIR, and the addition of phthalates or benzoates plasticisers by both ATR-FTIR and µ-Raman.

## 1. Introduction

Poly(vinyl acetate) (PVAc) was first introduced in 1912 [1], but for the first formulations an organic solvent was used. In the late 1940s, safer and non-toxic PVAc aqueous emulsions followed very quickly [2], and shortly after their availability, these plastic paints started to be used by artists, being now present in several artworks, museums, and collections worldwide.

Portuguese artists have used emulsions based on vinyl acetate at least since the early 1960s [3,4,5,6], having produced some of the most important paintings of the Portuguese art scene of their time. Joaquim Rodrigo (1912–1997) shows a consistent use of a vinyl binding medium until he painted his last work in 1990 [6]. Ângelo de Sousa (1938–2011), using the painting media as his instrument of plastic experimentation, was also one of the first Portuguese artists to have used vinyl-based paints [6]. Julião Sarmento (1948–2021), working since 1975, made use of different vinyl emulsions manufactured both by Portuguese and international companies [5].

However, whereas some paintings from the 1990s by Julião Sarmento made with a vinyl acetate-based (VAc-based) white glue are showing signs of degradation clearly evident by yellowing [5], paintings by the artists Ângelo de Sousa and Joaquim Rodrigo (produced since the 1960s), also made with a VAc-based white glue, so far, do not show clear signs of ageing [6].

PVAc, as a homopolymer, is considered a relatively stable material [7]. However, it has already been observed that some VAc-based emulsions (commonly known as white glues) are more unstable than the homopolymer [7], confirming that different vinyl formulations can behave differently over time. PVAc can deteriorate by multiple pathways: photooxidation, via main chain scission and formation of double bonds and other functional groups; emulsion hydrolysis; and/or via biodeterioration because VAc-based emulsions are highly bioreceptible and the biocides added for film protection can fail [8].

PVAc emulsions are obtained based on emulsion polymerisation by mixing the vinyl acetate monomer with water, a surfactant, and an initiator [9], resulting in the production of fine quality films: transparent, lightfast, with good hardness, gloss, and adhesive strength [10]. Still, to improve the optical, physical, and chemical properties of the final film, other additives can be incorporated. These additives include co-monomers, plasticisers, protective colloids, colorants, fillers, and stabilisers, among others [11,12], which can influence the film degradation by promoting different reactivities with the surrounding environment. Among these additives, plasticisers (sometimes up to 20% by weight) are of particular importance as these emulsions are known to form slightly hard and brittle films, requiring the addition of components capable of lowering its glass transition temperature. Phthalates, namely, dibutyl phthalate (DBP), are common plasticisers; however, being volatile they tend to migrate out of the paint film, having a high impact in its ageing behaviour. Another possibility is the use of internal plasticisers by the addition of softer monomers to form permanent flexible films. Some of the co-monomers used are n-butyl acrylate (nBA), 2-ethylhexyl acrylate (2-EHA), di-n-butyl maleate and C9-C10 branched vinyl esters (vinyl versatate), known under the trade name VeoVa [13]. For instance, the P(VAc-VeoVa) copolymer is considered to have better hydrolytic stability and UV resistance than the homopolymer or PVAc conventional copolymers [12,13].

Thus, the identification of each component in the VAc-based emulsion prior the understanding of its influence in the vinyl film ageing and the establishment of conservation or preventive strategies is fundamental.

So far, several studies have used different techniques for the molecular characterisation of VAc-based films and paints [7,14,15,16,17,18,19,20,21,22,23,24,25,26,27,28,29]. Within these studies, Fourier transform infrared spectroscopy (FTIR) [7,15,18,20,21,23,24,25,27,28,29] and pyrolysis–gas chromatography and mass spectrometry (Py–GC/MS) [14,15,16,17,21,22,23,26,27] have been highly used, but other techniques such as size exclusion chromatography (SEC) [7], atomic force microscopy (AFM) [19], scanning electron microscopy–energy-dispersive X-ray microanalysis (SEM/EDX) [20] and UV–Vis spectroscopy [20], differential scanning calorimetry (DSC) [18], thermo-gravimetric analysis (TGA) [18], mechanical properties test [18] have also been used, among others. Still, most of these studies have been individually focused on a narrow set of PVAc compositions (smaller variability) and/or on the use of Py-GC/MS, which is time-consuming, requires strong expertise and solid databases for an accurate interpretation, and is also less accessible to museums. Furthermore, Raman spectroscopy has not been addressed in those studies, even though its complementarity with infrared spectroscopy in the analysis of synthetic polymers has already been proved [30,31,32,33].

In this study, a broader set of VAc-based emulsions was gathered, including PVAc homopolymers and copolymers with ethylene, acrylic and vinyl versatate monomers, as well as plasticised and non-plasticised formulations, and also formulations stabilised with poly(vinyl alcohol) (PVAl). Films from these emulsions have been analysed by in situ and quick data acquisition techniques such as infrared spectroscopy in attenuated total reflection (ATR-FTIR) and micro-Raman (µ-Raman) spectroscopies. The main goal is to propose a more accessible analytical methodology for the characterisation of a wide range of vinyl emulsion films, aiming at detecting several components in these composite formulations based on the identification of spectral markers.

This investigation is also part of the research project ‘Plastic Paints in Art: the impact of manufacturing processes on their long-term stability’, taking place at the Department of Conservation and Restoration from NOVA School of Science and Technology. It aims at contributing to a second part of the project focused on the establishment of a correlation between the emulsion formulation and its long-term stability studied by artificial ageing using light as the trigger and accelerator.

## 2. Materials and Methods

### 2.1. Samples

Vinyl homopolymer emulsions with known formulations: three different vinyl emulsion formulations have been prepared following the recipe in [34]: (i) pure PVAc emulsion, (ii) PVAc emulsion containing 5% of poly(vinyl alcohol), and (iii) PVAc emulsion containing 10% of poly(vinyl alcohol). These three emulsions are non-plasticised formulations and the poly(vinyl alcohol) was added as an emulsion stabiliser, which also contributes to lowering the film Tg. Reagents were bought from Sigma-Aldrich (St. Louis, MO, USA).

Commercial vinyl emulsions with known and unknown formulations: twelve different vinyl emulsion formulations have been collected from: the industrial company Resiquímica—Chemical Resins (Sintra, Portugal), and art supply stores (Casa Varela—Artes Plásticas (Lisbon, Portugal), an old art supplier selling from the Portuguese producer A Favrel Lisbonense (Lisbon, Portugal), and Casa Ferreira—Artigos para Belas Artes (Lisbon, Portugal)). The known formulations result from information collected on technical data sheets (Resiquímica—Chemical Resins) and on research studies previously carried out [5,6,7]. Information on the Favrel company can be found in [6].

A summary of the known information regarding the selected vinyl acetate-based emulsions composition can be found in Table 1. Before analysis, the selected emulsions were applied with a thickness of 200 µm to glass plates and left to dry at room temperature for several weeks.

### 2.2. Methodology

For the entire set of analyses, each film was analysed in three different areas by each technique, one spectrum per area. Infrared spectroscopy in attenuated total reflection (ATR-FTIR) and micro-Raman spectroscopy were selected because of the possibility of conducting analyses in situ, without any sampling or sample preparation. Another aspect taken into consideration is the complementarity of the two techniques, which can be determinative in the attempt of a complete identification of the several compounds present in such composite formulations. Although Raman microscopy has not been commonly applied in characterisation studies of VAc-based emulsions, it has already proved to be a valuable technique for the study of polymers and polymerisation processes [30,31,32] as well as on the ageing of polyurethane foams [33]. The high sensitivity of Raman microscopy to polymer conformational structures and to C–C and C=C stretching vibrations are some of the reasons pointed for that use [30,31,32,33]. On the other hand, infrared spectroscopy is more sensitive in detecting small polar molecules and single bonded chemical compounds such as C–C, C–O and C–H. Therefore, both techniques are used in this study for the identification of characteristic bands and spectral profiles (here named as spectral markers) for each component in the vinyl emulsion.

### 2.3. Instrumentation

Infrared spectroscopy in attenuated total reflection (ATR-FTIR) was carried out with the Handheld Agilent 4300 spectrophotometer (Agilent, Santa Clara, CA, USA), equipped with a ZnSe beam splitter, a Michelson interferometer, and a thermoelectrically cooled DTGS detector. Spectra were acquired with a diamond ATR module, 128 scans and 4 cm^−1^ resolution, between 4000 and 650 cm^−1^. Background spectra was collected between every acquisition. The OriginPro 8 software (OriginLab Corporation, Northampton, MA, USA) was used for the analysis of the spectra.

Raman microscopy was carried out using a Horiba Jobin Yvon LabRAM 300 spectrometer (Kyoto, Japan), equipped with a He-Ne laser 17 mW operating at 632.8 nm and coupled to the Confocal Microscope with high stability Olympus BX41. The system was calibrated to better than 1 cm^−1^ using a silicon standard before the measurements. The laser beam was focused on the sample by a ×100 objective lens to give a spot size of approximately 1 μm. The laser power at the surface of the samples was controlled using neutral density filters. Grating of 1800 groves/mm was used to collect spectra between 300 and 1800 cm^−1^, and 2700 and 3800 cm^−1^. Spectra were recorded as an extended scan. Raman data analysis was performed using LabSpec 5 software (Horiba, Kyoto, Japan) and all spectra were baseline-corrected and normalised for comparison reasons.

## 3. Results and Discussion

The following sections discuss the identification of each component in the vinyl acetate-based emulsion based on ATR-FTIR and micro-Raman spectroscopies.

### 3.1. PVAc and PVAl Spectral Markers

**Figure 1:**
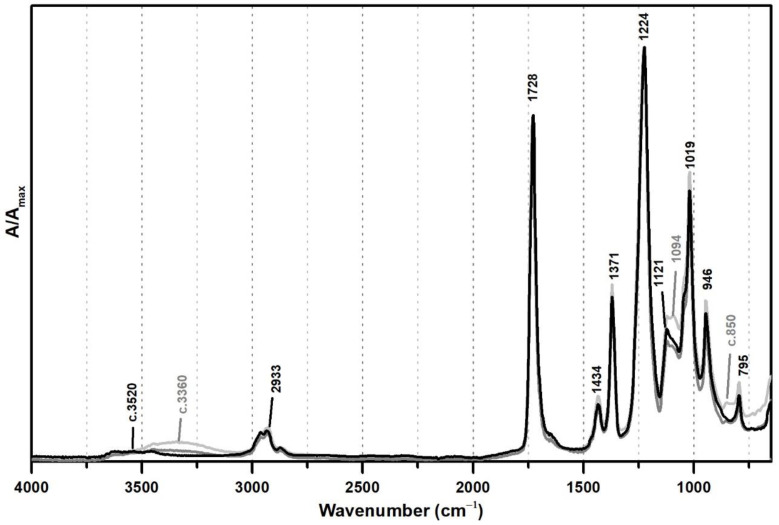
Infrared spectra in ATR of vinyl emulsion films produced for the purpose of this study: pure PVAc (black) and PVAc stabilised with 5% (dark grey) and 10% (light grey) of PVAl.

From Figure 1, the spectral changes related to the addition of PVAl as a protective colloid (emulsifier, stabiliser) to the PVAc emulsion are significant, even though this additive was added at percentages such as 5% and 10%. The weak band previously centred at ~3520 shifts to ~3360 cm^−1^ due to an increase of OH groups in the emulsion from the presence of PVAl (Figure 1), and there is the appearance of two shoulders at 1094 and ~850 cm^−1^, assigned to C–O and C–C–O stretching [35], respectively.

**Figure 2:**
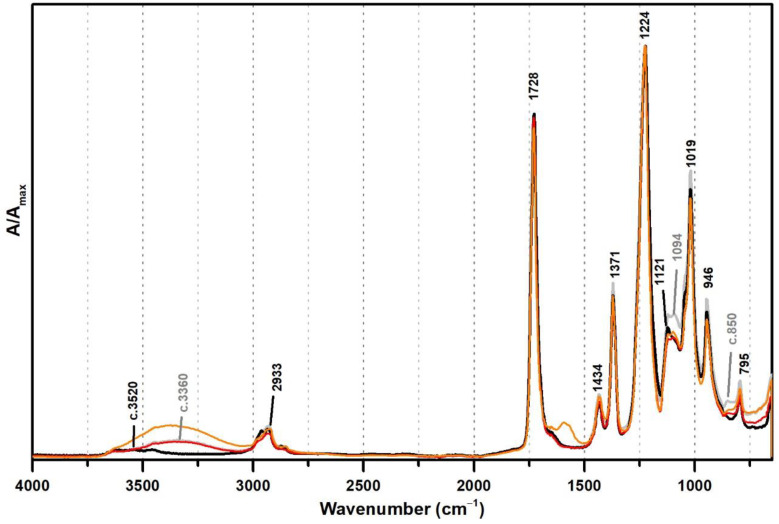
Infrared spectra in ATR of the vinyl acetate emulsion films: pure PVAc (black), PVAc stabilised with 10% of PVAl (grey), Polidisp 1080 (red), and Polidisp DH (orange). According to technical data sheet, the Polidisp emulsions in these spectra are composed of PVAc stabilised with PVAl.

These PVAl markers were also detected in the ATR-FTIR spectra of two commercial formulations (Polidisp DH and Polidisp 1080) of vinyl acetate-based emulsions stabilised with PVAl, as described in technical datasheets (Figure 2). However, Polidisp DH spectrum shows a higher relative intensity of the OH stretching band (here centred at c. 3370 cm^−1^) than both references and Polidisp 1080. As the references and Polidisp 1080 are more recent emulsions (Table 1), this increase might be associated to the release of an acid odour from the emulsion recipient, which is possibly related to the formation of acetic acid as an ageing product. The increase in OH groups together with a small increase in intensity of the shoulder at ~1650 cm^−1^ (C=O stretching of carboxylic acids [36]) and the appearance of a weak band with maximum at ~1594 cm^−1^ (C=C stretching possibly related to –CH=CH– species formed during PVAc degradation [37]), suggest the formation and release of acetic acid [20,23] as detected by its characteristic odour.

Regarding Raman spectroscopy, no spectral markers were detected for the addition of PVAl as a protective colloid in the PVAc emulsion. This was not unexpected as Raman is more sensitive to symmetric and apolar bonds [30,31,32]. As PVAl is rich in hydroxyl groups, the absence of evident spectral differences between these formulations in Raman spectroscopy was already expected.

### 3.2. VeoVa Spectral Markers

**Figure 3:**
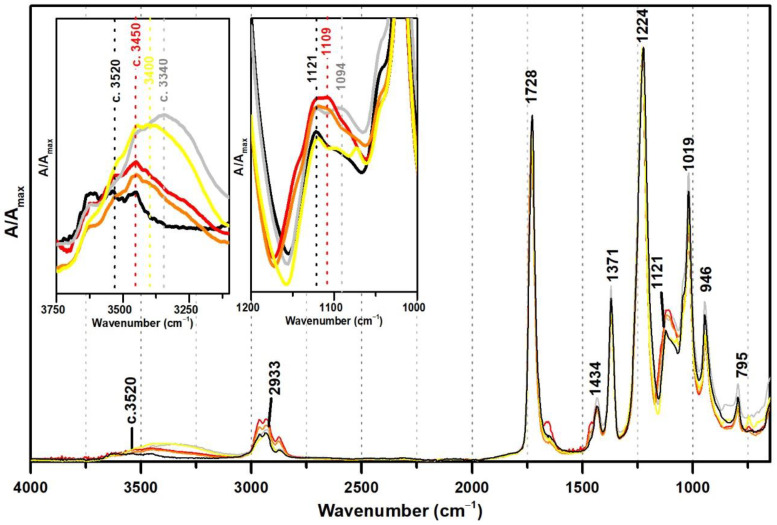
Infrared spectra in ATR of the pure PVAc emulsion (black), PVAc stabilised with 10% PVAl (grey), Polidisp DM21 (red), Polidisp DM23 (orange), and Imofan AV44/11 (yellow). Insets: details between 3750–3100 cm^−1^ and between 1200 and 1000 cm^−1^. According to technical data sheets, Polidisp DM21 and DM23 are composed of P(VAc-VeoVa), and according to [5], Imofan AV44/11 is composed of P(VAc-VeoVa) plasticised with DiBP.

Figure 3 shows the ATR-FTIR spectra of both references of PVAc and PVAc stabilised with PVAl (10%), as well as the spectra of three commercial formulations of vinyl acetate copolymers with VeoVa (Polidisp DM21, Polidisp DM23, and Imofan AV44/11). VeoVa is commonly added to vinyl acetate-based emulsions as an internal plasticiser (lowering the Tg). As shown in Figure 3, no evident spectral markers for this internal plasticiser were detected as already suggested by [6], even though micro-FTIR (in transmission mode) was used in that study. However, a small shift of the OH band (previously centred at 3520 cm^−1^) to c. 3450 cm^−1^ is observed when VeoVa is present in the formulation (Figure 3). Even though no clear correspondence can be found between this shift and the molecular structure of VeoVa (absence of OH groups), this shift may be associated to a change in the polarity of the polymer resulting in a slightly spectral change in the IR.

Furthermore, the Imofan AV44/11 spectrum shows a higher shift to lower frequencies (3400 cm^−1^) than the PolidispDM21 and PolidispDM23 spectra (centred at 3450 cm^−1^). This might be explained by a possible stabilisation of Imofan AV44/11 with PVAl as in addition to this shift, the shoulders at 1094 and 850 cm^−1^ are found.

**Figure 4:**
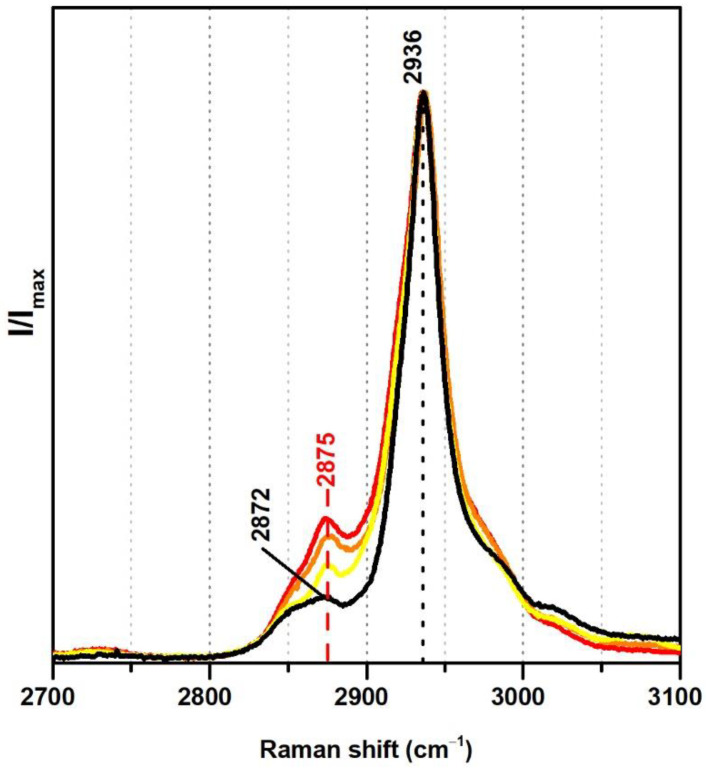
Raman spectra of the pure PVAc emulsion (black), Polidisp DM21 (red), Polidisp DM23 (orange), and Imofan AV44/11 (yellow). According to technical data sheets, these Polidisp commercial formulations are composed of P(VAc-VeoVa) and, according to the work in [5], the Imofan AV44/11 is composed of P(VAc-VeoVa) plasticised with DiBP.

From Raman spectroscopy it was not possible to detect significant differences regarding the addition of VeoVa in the region 300–1800 cm^−1^. On the other hand, in the region between 2700 and 3100 cm^−1^ a spectral marker might be suggested, namely, a more pronounced band at ~2875 cm^−1^ (Figure 4). In fact, in the presence of VeoVa, the ratio between the intensities of the band at ~2875 cm^−1^ and the maximum band at 2936 cm^−1^ doubles: without VeoVa (≈0.1) and with VeoVa (≈0.2) (Table 2). As VeoVa shows the same functional groups as the vinyl acetate monomer (C=O and C–O–C) but varies more significantly in a larger number of CH_2_ groups in its side chain, the attribution of the band at ~2875 cm^−1^ as a spectral marker may gain strength, as this band is assigned to CH_2_ bonds [38,39] and its intensity increases in the presence of emulsions with VeoVa as a co-monomer. Still, these two VeoVa markers (from IR and Raman) should not be independently used since only one minor spectral change was detected per technique.

### 3.3. Plasticisers Spectral Markers

**Figure 5:**
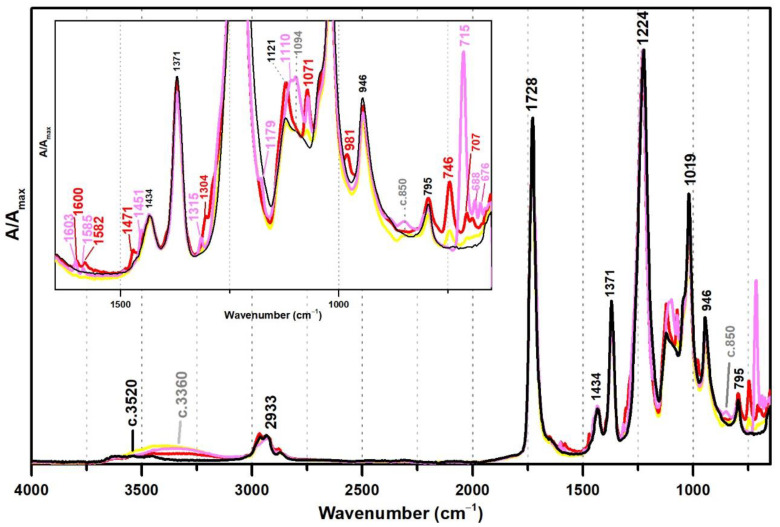
Infrared spectra in ATR of the pure PVAc emulsion (black), Bizonte (pink), Vulcano V7 (red) and Imofan AV44/11 (yellow). Inset: detail between 1650 and 650 cm^−1^. According to the authors of [5], Bizonte is composed of PVAc plasticised with DPGDB and DEGDB, Vulcano V7 is composed of PVAc plasticised with DiBP, and Imofan AV44/11 is composed of P(VAc-VeoVa) plasticised with DiBP.

In this section, VAc-based emulsions plasticised with diisobutyl phthalate (DiBP), dipropylene glycol and diethylene glycol dibenzoates (DPGDB and DEGDB) were used to include two common families of plasticisers in the history of the vinyl paint industry (covering oldest and more recent formulations). For their detection, even though ATR-FTIR and micro-Raman spectroscopies proved to be effective, Raman showed a higher detection limit, particularly useful for cases showing low concentration of plasticiser and/or less plasticiser migration to the surface. This advantage is probably related to the higher resolution of the Raman equipment (micro-scale resolution with a spot size of c. 1 μm compared to ~200 µm of ATR-FTIR) and to a more surface analysis carried out by ATR (very small penetration depth of the IR beam, ~2 µm). An example of this situation was observed for Imofan AV44/11 where its IR spectrum did not clearly show the presence of plasticisers as Vulcano V7, both plasticised with DiBP (Figure 5). While Vulcano V7 clearly shows characteristic bands of phthalates at 1650–1450 cm^−1^ (with very weak intensity) and 800–650 cm^−1^ (with weak to medium intensity), Imofan AV44/11 does not show any band in the first region (1650–1450 cm^−1^), and only one very weak band is observed at 745 cm^−1^, which might not be interpreted as a marker for the DiBP. However, from the careful analysis of these three spectra (Bizonte, Vulcano V7, and Imofan AV44/11) (Figure 5), it might be possible to propose the presence of a small-medium band at c. 1071 cm^−1^ (C-O stretching) as indicator of plasticisers in the formulation because it can be found in all spectra. Then, the additional presence of bands at 746 and 707 cm^−1^ are indicators of DiBP, and a more intense band at 715 cm^−1^ along with weaker bands at 688 and 676 cm^−1^ indicate the presence of DPGDB and DEGDB [36,40]. From Figure 5, it is also possible to mention that the region between 800 and 650 cm^−1^ in the IR spectrum is the most beneficial in distinguishing the two families of plasticisers: phthalates and benzoates. This observation can be justified by a higher penetration depth of the IR beam at lower frequencies (in ATR mode), promoting a higher intensity of the bands in this region (especially when compared to micro-FTIR, transmission mode) and to the common absorption of aromatic substituents at these frequencies. Furthermore, it may be possible to also indicate a stabilisation of Bizonte with PVAl as the shift of the O–H stretching band to 3360 cm^−1^ is detected, as well as the weak bands at approximately 1094 and 850 cm^−1^ (as in Imofan AV44/11).

**Figure 6:**
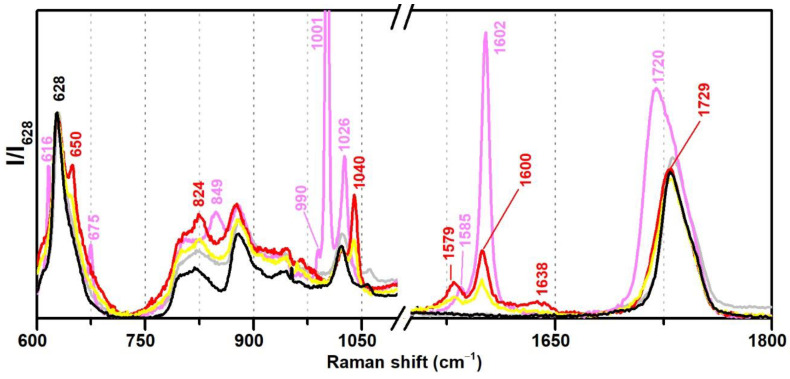
Raman spectra of the pure PVAc emulsion (black), Bizonte (pink), Vulcano V7 (red), and Imofan AV44/11 (yellow) between 600–1800 cm^−1^. According to the authors of [5], Bizonte is composed of PVAc plasticised with DPGDB and DEGDB, Vulcano V7 is composed of PVAc plasticised with DiBP, and Imofan AV44/11 is composed of P(VAc-VeoVa) plasticised with DiBP.

Regarding Raman spectroscopy, the regions between 600–700, 950–1100, and 1550–1650 cm^−1^ were identified as the most important for the discrimination between plasticised and non-plasticised formulations (Figure 6). Furthermore, it was very easy to distinguish phthalates (such as DiBP) and benzoates (DPGDB and DEGDB). VAc-based emulsions plasticised with DiBP show Raman bands at 650, 1040, 1579 and 1600 cm^−1^ of weak to medium intensity, whereas DEGDB and DPGDB show bands at 616, 675, 849, 1001, 1602, and 1720 cm^−1^ with medium to very strong intensities, being the band at 1001 cm^−1^ one of its strongest markers in Raman spectroscopy.

### 3.4. Other Compounds

Unfortunately, no spectral markers were possible to identify for the ester of acrylic acid as a co-monomer. This could be justified by (i) the presence of this monomer in a limited number of samples (only two commercial formulations with known formulations, as terpolymers with VAc and VeoVa), (ii) to the presence of moieties and functional groups like the other monomers, and/or (iii) to a low ratio of this monomer in the formulation, especially when compared to VAc (main component) and VeoVa, in terpolymer emulsions.

**Figure 7:**
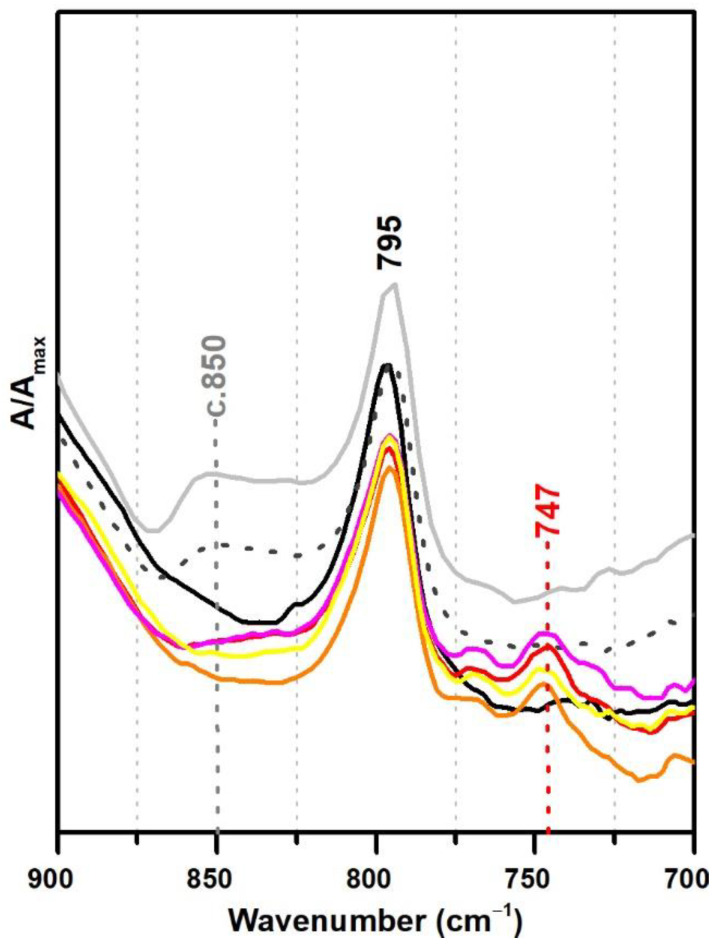
Infrared spectra in ATR spectra of the pure PVAc emulsion (black), PVAc stabilised with 10% PVAl (light grey), Polidisp 1080 (dotted dark grey), Polidisp DM22 (red), Polidisp DM9725 (orange), Polidisp DM 21 (pink), and Polidisp DM23 (yellow). According to technical data sheets, only Polidisp DM21, DM22, DM23, and DM9725 are stabilised with “polymeric compounds”, as referred by the company.

For the polymeric compounds frequently addressed in the technical datasheets from Resiquímica as stabilising additives for vinyl-acetate based emulsions (Polidisp DM21, DM22, DM23 and DM9725), a spectral marker in ATR-FTIR was possibly detected at ~747 cm^−1^ (Figure 7), even though the chemical nature of these polymeric compounds was not identified. Nonetheless, as the band was only detected in such formulations—being absent in all emulsions produced for the purpose of this study and for the ones identified as stabilised with PVAl—this spectral marker might be used to alert to a different type of emulsion stabilisation rather than PVAl. Nevertheless, a careful analysis of the spectra needs to be carried out as DiBP also shows a band at 745 cm^−1^ (even though the identification of DiBP requires the presence of other markers).

**Figure 8:**
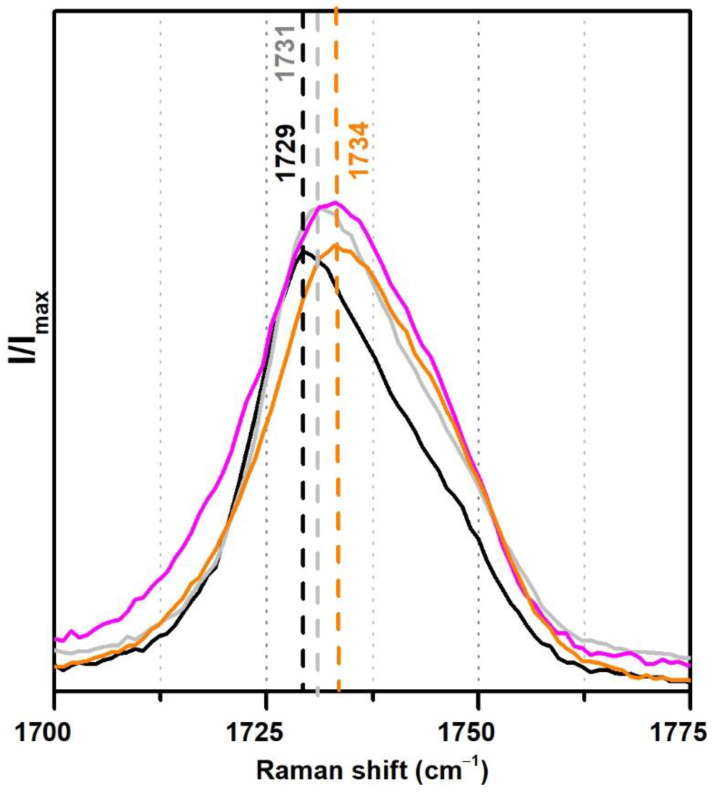
Raman spectra of the pure PVAc emulsion (black), PVAc stabilised with 10% PVAl (grey), Polidisp DM21 (pink), Polidisp DM23 (orange) between 1700 and 1775 cm^−1^. According to technical data sheets, these Polidisp commercial formulations are composed of P(VAc-VeoVa) stabilised with polymeric compounds.

From the analysis of the Raman spectra (Figure 8), the observed shift of the carbonyl band from 1729 to 1734 cm^−1^ in the case of the two Polidisp DM21 and DM22 may also be due to a stabilisation with polymeric compounds other than PVAl.

### 3.5. Proposing Spectral Markers for Vinyl Acetate-Based Emulsions

From the collected data, spectral markers are proposed for several components in the vinyl acetate-based emulsion, summarised in Table 3 and Table 4. These markers include monomers, stabilisers, and plasticisers in the composite formulation.

### 3.6. Proposing Compositions for the Unknown Formulations

**Figure 9:**
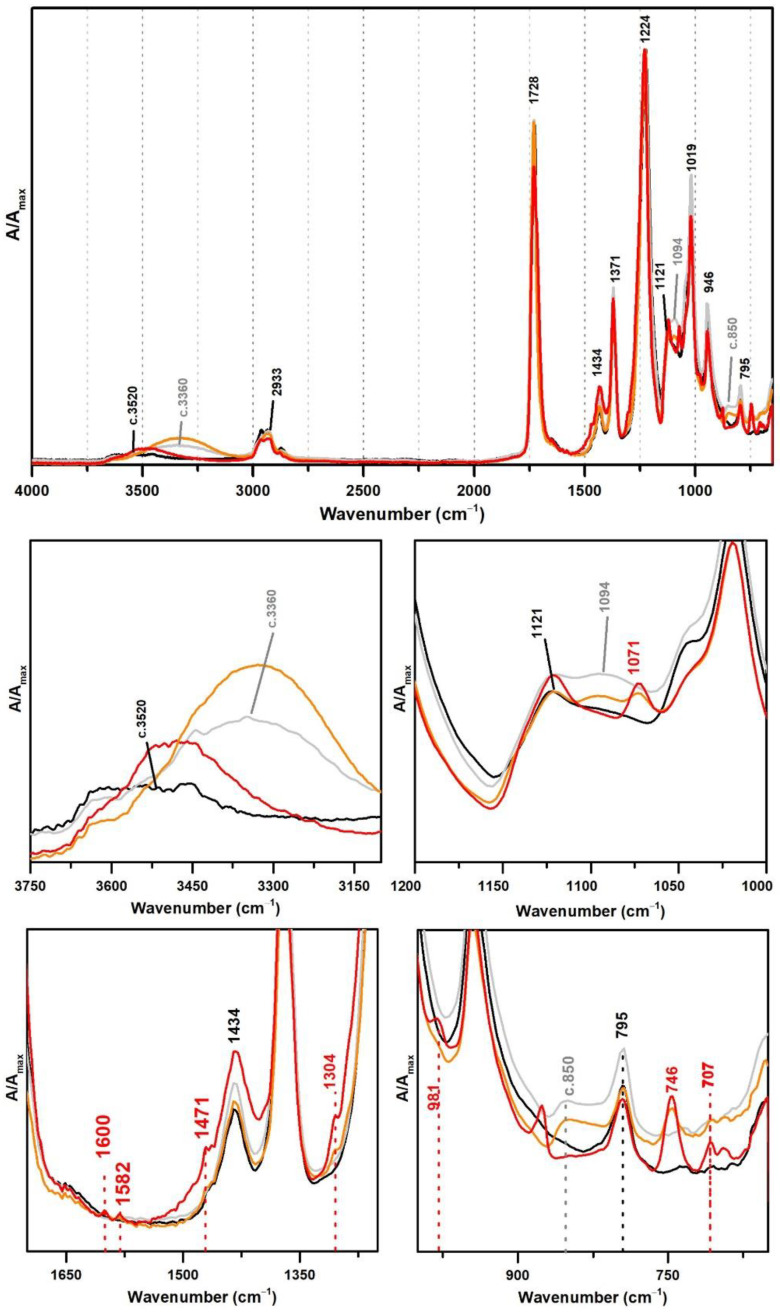
Infrared spectra in ATR of the pure PVAc emulsion (black), PVAc stabilised with PVAl (10%) (grey), Giotto Vinilik White PVA Glue (orange) and V2 Cola Tudo Incolor (red).

From Figure 9 and Table 3 and Table 4, it might be possible to propose Giotto Vinilik as a PVAc-based emulsion stabilised with PVAl and plasticised with DiBP (also indicated by Raman), whereas V2 is probably a P(VAc) plasticised with DiBP (also confirmed by Raman).

## 4. Conclusions

For the first time, a large set of Vac-based emulsions (15 different compositions) were gathered for the identification of different components in the formulation. This set included homopolymers and copolymers, plasticised and non-plasticised formulations, artistic and industrial brands, as well as known and unknown compositions. The identification of the components was carried out based on in situ analysis by infrared and Raman spectroscopies, exploring the potential of the two techniques in the detection of spectral markers for the identification of the polymer and additives.

The availability of a large variety of vinyl acetate-based emulsions for artists was reinforced as both homopolymers and copolymers have been detected in well-known brands in the Portuguese context (such as Vulcano, Bizonte, V2, and Giotto), as well as the presence of different emulsion stabilisers and plasticisers which may dictate different life spans for such artworks. This study also alerted for the importance of establishing straightforward analytical protocols capable of identifying the components in a vinyl emulsion, supported by techniques commonly available in museums and conservation labs such as infrared and Raman spectroscopies. Even though a rigorous identification of minor components might only be possible with gas chromatography and mass spectrometry coupled to different devices, this technique requires sampling, is time-consuming in both performance and results interpretation, and demands strong expertise and databases for an accurate peak assignment.

Moreover, the combined use of infrared and Raman spectroscopy proved to be very useful as different spectral markers were detected by each technique, depending on its sensitivity and detection limits, confirming their complementarity. In addition, besides the clear identification of vinyl acetate-based emulsions based on ATR-FTIR and Raman, it was also possible to propose spectral markers for its copolymers with the monomer vinyl versatate (VeoVa, by Raman), the stabilisation of the emulsion with poly(vinyl alcohol) (PVAl, by ATR-FTIR), and the addition of phthalates or benzoates as plasticisers (by both ATR-FTIR and Raman). In sum, ATR-FTIR was more useful for the detection of PVAl as a stabiliser, whereas Raman was more efficient in the identification of plasticisers.

## Figures and Tables

**Table 1:** Summary of the collected compositional information of the vinyl acetate-based emulsions before the current study.

PVAc Aqueous Emulsion	Reference/Commercial Name	Date of the Emulsion Acquisition/Production	Viny Emulsion Composition
Homopolymer	Vinyl Emulsion A	2018	Pure PVAc, non-plasticised ^a^
	Vinyl Emulsion B	2018	PVAc stabilised with 5% of PVAl, non-plasticised ^a^
	Vinyl Emulsion C	2018	PVAc stabilised with 10% of PVAl, non-plasticised ^a^
	Polidisp DH	2000s *	PVAc stabilised with PVAl, non-plasticised ^b^
	Vulcano V7	2004 **	PVAc + DiBP (≈21% of plasticiser is suggested) ^c^
	Bizonte	2009 **	PVAc + DPGDB and DEGDB ^c^
	Polidisp 1080	2017 *	PVAc stabilised with PVAl, non-plasticised ^b^
Copolymer	Imofan^®^ AV 44/11	2009 ^##^	P(Vac-VeoVa) + DiBP ^c^
	Polidisp DM 21	2000s *	P(VAc-VeoVa) stabilised with polymeric compounds, non-plasticised ^b^
	Polidisp DM 23	2000s *	P(VAc-VeoVa), emulsified with surfactants and stabilised with polymeric compounds ^b^
	Polidisp DM 22	2000s *	P(VAc-VeoVa-ester of acrylic acid) stabilised with polymeric compounds ^b^
	Polidisp DM 9725	2000s *	P(VAc-VeoVa-ester of acrylic acid), emulsified with surfactants and stabilised with polymeric compounds ^b^
	Vinamul^®^ 3469	2009 ^#^	P(VAc-VC-E) (≈16% of PVC), non-plasticised ^c^
Unknown	V2 Cola Tudo Incolor	2000s ***	Unknown
	Giotto Vinilik PVA Glue	2000s ^###^	Unknown

^a^: produced for the purpose of the study; ^b^: according to *Resiquímica* technical datasheets; ^c^: according to [5]. * obtained directly at the Portuguese manufacturing company *Resiquímica-Chemical Resins*; ** purchased from *Casa Varela-Artes Plásticas*; *** purchased from *Casa Ferreira–Artigos para Belas Artes*; ^#^ obtained directly at the Portuguese distributor *Globalcor, S.A.*; ^##^ obtained directly at the Portuguese distributor *Sarcol*; ^###^ supplied by a private owner.

**Table 2:** Raman intensity ratios for the symmetric and antisymmetric CH_2_ bonds in vinyl-acetate based emulsions with and without VeoVa in the formulation.

Vinyl-Acetate Based Emulsions	Raman Spectra	I_2875_/I_2936_	Average
without VeoVa	PVAc	0.106 ± 0.002	0.101
	PVAc stabilised with 10%PVAl	0.103 ± 0.007	
	PVAc stabilised with PVAl, *Polidisp 1080*	0.093 ± 0.003	
with VeoVa	P(VAc-VeoVa), *Polidisp DM21*	0.246 ± 0.001	0.206
	P(VAc-VeoVa), *Polidisp DM23*	0.219 ± 0.007	
	P(VAc-VeoVa), *Imofan AV44/11*	0.153 ± 0.014	

**Table 3:** Suggested spectral markers in ATR-FTIR and assignment for several components of the studied vinyl acetate-based emulsions.

PVAc	PVAc Stabilised w/PVAl	PVAc Stabilised w/“Polymeric Compounds” of Unknown Composition	PVAc Plasticised w/DiBP	PVAc Plasticised w/DPGDB and DEGDB	VAc Copolymer w/VeoVa	Assignment	Refs.
max. at c. 3520 (w, br)	**max. at c. 3360** (w, br)				max. at c. 3450 (w, br)	*ν* (OH)	[23,35,38]
**2933 (w)**						*ν*_as_ (C–H_2_)	[21,27,35]
**1728**–**1730** (vs)						*ν*_s_ (C=O)	[21,27,28]
			*1600 (vw)*	*1603 (vw)*		aromatic ring skeletal vibration	[41]
			*1582 (vw)*	*1585 (vw)*		aromatic ring skeletal vibration	[41]
			*1471 (sld)*	*1451 (sld)*		*δ*_as_ (C–H_3_)/*δ*(C–H_2_)	
**1371** (m)						*δ*_s_ (C-H_3_)	[21,27]
			*1304 (sld)*	*1315 (vw)*		*ν_as_* (ring)	
**1224** (vs)						*ν* (C–O) *ν* (C–C)	[21,27]
				*1179 (sld)*		*ν*_s_ (CH–O)	
1121 (m)						*ν* (C–O) *ν* (C–C)	[21,27,28]
	**c. 1094 (sld)**					*ν* (C–O)	[35,42]
				***1110 (sld)***		*ν_as_* (C-O-CO)	
			**1071 (m)**	**1071 (m)**		*ν* (C–O)	[43]
**1019** (s)						*ν* (C–O) *ν* (C–C) *ν_s_* (CH–O)	[21,27,28]
			**981 (sld)**				
**946** (m)						*δ* (C–H) *ν* (C–C–O)	[21,27,28,35]
	**c. 850 (sld)**					*δ* (C–H_2_) *ν* (C–C–O)	[35,42]
**795** (w)						*δ* (C–H)	[21,27]
		**747 (vw)**	**746 (w)**			*δ* (C–H) aromatic out of plane	[40]
				**715 (m)**		*δ* (C=O) out-of-plane wag and aryl *δ* (C–H) wag	[36]
			**707 (vw)**			*	
				**688 (vw)**		*	
				**676 (vw)**		*	

PVAc: refers to poly(vinyl acetate), PVAl: poly(vinyl alcohol), DiBP: diisobuty phthalate, DPGDB: dipropylene glycol dibenzoate, DEGDB: diethylene glycol dibenzoate, and VeoVa: vinyl versatate. *ν*—stretching vibration; *δ*—deformation vibration; vs—very strong; s—strong; m—medium; w—weak; vw—very weak; br—broad; sld—shoulder. Frequencies marked in italic are spectral markers that may not always be present, probably depending on the concentration of the compound in the formulation or the emulsion ageing which may lead to the migration of the plasticisers. Frequencies marked in bold are the best spectral markers, independently on the compound concentration or film ageing. * —assignment not found.

**Table 4:** Suggested spectral markers in Raman and assignment for several components on the studied vinyl acetate-based emulsions.

PVAc	PVAc Plasticised w/DiBP	PVAc Plasticised w/DPGDB and DEGDB	VAc Copolymer w/VeoVa	Assignment	Refs.
628 (vs)				*δ* (C–H_2_)	[38]
880 (s)				*ν* (C–C)	[38]
		1001 (vs)			[44]
1021 (s)		1026 (s)		δ (CH_2_)	[38]
	1040 (s)				[44]
1128 (m)					[38]
		1277 (s)			[44]
1357 (m)				δ_s_ (C–H_2_)	[38]
1372 (m)				δ_as_ (C–H_3_)	[38]
1439 (s)					[38]
	1579 (w)				[44]
		1584 (w)			[44]
	1599 (m)			ν (C=C) arom.	[44]
		1602 (vs)		ν (C=C) arom.	[44]
	1639 (vw)				[44]
1729 (s)		1720 (s)		ν (C=O)	[38]
2872 (sld)			2875 (m)	ν_s_ (C–H_2_)	[38]
2936 (s)				ν_as_ (C–H_2_)	[38]
		3072 (m)		ν (=C–H) arom.	[44]

PVAc: refers to poly(vinyl acetate), PVAl: poly(vinyl alcohol), DiBP: diisobuty phthalate, DPGDB: dipropylene glycol dibenzoate, DEGDB: diethylene glycol dibenzoate, and VeoVa: vinyl versatate *ν*—stretching vibration; *δ*—deformation vibration; vs—very strong; s—strong; m—medium; w—weak; vw—very weak; br—broad; sld—shoulder.

## Data Availability

Not applicable.
